# Stereotactic body radiation therapy for palliative management of pancreatic adenocarcinoma in elderly and medically inoperable patients

**DOI:** 10.18632/oncotarget.24713

**Published:** 2018-03-27

**Authors:** John F. Ryan, Lauren M. Rosati, Vincent P. Groot, Dung T. Le, Lei Zheng, Daniel A. Laheru, Eun J. Shin, Juan Jackson, Joseph Moore, Amol K. Narang, Joseph M. Herman

**Affiliations:** ^1^ Department of Radiation Oncology and Molecular Radiation Sciences, Sidney Kimmel Comprehensive Cancer Center, The Johns Hopkins University School of Medicine, Baltimore, Maryland, USA; ^2^ Department of Surgery, The Johns Hopkins University School of Medicine, Baltimore, Maryland, USA; ^3^ Department of Oncology, The Johns Hopkins University School of Medicine, Baltimore, Maryland, USA; ^4^ Department of Radiation Oncology, The University of Texas MD Anderson Cancer Center, Houston, Texas, USA

**Keywords:** stereotactic body radiation therapy (SBRT), palliative care, pancreatic cancer, radiation, elderly

## Abstract

Stereotactic body radiation therapy (SBRT) represents a promising treatment option for patients with localized pancreatic ductal adenocarcinoma (PDAC) who cannot tolerate surgical therapy. We retrospectively reviewed the records of patients with localized PDAC treated with SBRT at our institution between 2010 and 2016 to identify patients deemed medically inoperable due to poor performance status, advanced age, and/or comorbid conditions. Overall survival (OS), progression-free survival (PFS), and local progression-free survival (LPFS) were estimated using Kaplan-Meier curves. Twenty-nine patients were included. Median age was 74 (IQR 68-79). Thirteen patients (45%) had an Eastern Cooperative Oncology Group performance status of 2. Six patients (19%) had chronic obstructive pulmonary disease, 9 (31%) had cardiovascular disease, and 17 (58%) had diabetes mellitus. SBRT was delivered over 5 fractions to a median dose of 28 Gy (IQR, 25-33). Twenty-two patients (76%) received induction chemotherapy prior to SBRT, and 9 (31%) received maintenance chemotherapy after SBRT. Median OS was 13 months from diagnosis. Median OS and PFS were 8 and 6 months from SBRT, respectively. Six and 12-month LPFS rates were 91% and 78%, respectively. Patients receiving induction chemotherapy had superior survival from diagnosis than those who did not (14 vs. 7 months, p = 0.01). Three patients (10%) experienced acute grade ≥3 toxicity, and 1 patient (4%) experienced grade ≥3 late toxicity. Symptom relief was achieved at three-month follow-up in 8 of 11 patients (73%) experiencing abdominal pain. These results suggest SBRT may be safe and effective for patients who cannot tolerate surgery.

## INTRODUCTION

In the United States in 2016, an estimated 53,070 patients were diagnosed with pancreatic ductal adenocarcinoma (PDAC) and 41,780 died of the disease [1]. For patients with PDAC, definitive treatment involving surgical resection for non-metastatic localized disease remains the only potentially curative option. A subset of patients (20-30%) with localized disease, however, are not candidates for surgical therapy due to preexisting comorbid conditions, poor performance status, and/or advanced age [2].

PDAC is more common in the elderly as more than 66% of patients are aged 65 or older at diagnosis, with a median age of 70 [3]. The increased incidence of comorbid conditions in elderly patients may lead to poor performance status and reduced survival outcomes while precluding the possibility of curative-intent multimodality treatment including surgery, chemotherapy, and radiotherapy [4, 5]. Patients deemed ineligible for surgery are often offered either palliative single-agent chemotherapy or supportive care. Therefore, these patients with limited treatment options have poor survival and may die from painful local and systemic disease progression [6]. For patients who cannot tolerate surgery for localized PDAC, new tumor-directed treatment alternatives offering improved survival and quality of life are needed.

Stereotactic body radiation therapy (SBRT) is a promising, minimally invasive treatment that can result in local control, symptom palliation, and possibly extended survival outcomes as a primary treatment with an acceptable toxicity profile with similar survival and toxicity outcomes when delivered with both Cyberknife and linear accelerator-based approaches [7–13]. There is, however, a paucity of data regarding the application of SBRT in treating patients deemed medically inoperable. Two small retrospective studies have reported that SBRT may be a feasible and safe treatment for elderly patients with medical comorbidities and may provide relief of abdominal pain in as many as 79% of patients [14, 15]. However, more data are required to evaluate the efficacy, safety, and palliative capacity of SBRT in this unique patient population and to identify factors predictive of patient outcomes.

In this study, we report on the outcomes of patients with localized PDAC who were treated with SBRT at our institution after being deemed medically inoperable due to advanced age, medical comorbidities, and/or poor performance status.

## RESULTS

### Patients

Twenty-nine patients were identified who met inclusion criteria. Baseline clinicopathologic characteristics are summarized in Table 1. Median age was 74 (interquartile range [IQR], 68-79). Six patients (21%) had chronic obstructive pulmonary disease, 9 (31%) had cardiovascular disease, 17 (59%) had diabetes mellitus, 18 (62%) had hypertension, and 6 (21%) had a history of another cancer. Eleven patients (38%) had an Adult Comorbidity Evaluation-27 score of 2 (moderate), and 13 (45%) had a score of 3 (severe). Thirteen patients (45%) had an Eastern Cooperative Oncology Group performance status (ECOG PS) of 2, while the remaining 16 patients had an ECOG PS of 0 or 1 (55%). Those patients with an ECOG PS of 0 or 1 were considered medically inoperable due to their comorbid conditions and advanced age.

**Table 1:** Clinicopathologic characteristics

*Clinicopathologic variables*	*All patients (n=29)*
Gender, *n* (%)	
Male	11 (38%)
Age (years)	
Median (IQR)	74 (68-79)
Pre-SBRT CA19-9 (U/ml)	
Median (IQR)	161.6 (34.3-420.7)
≤37 U/ml, *n* (%)	7 (33%)
>37 U/ml, *n* (%)	20 (54%)
CA19-9 data unavailable	2 (13%)
Medical comorbidities, *n* (%)	
COPD	6 (19%)
Cardiovascular disease	9 (31%)
Diabetes mellitus	17 (58%)
Hypertension	18 (62%)
History of other cancer	6 (21%)
ECOG PS prior to SBRT, *n* (%)	
ECOG PS 0-1	16 (55%)
ECOG PS 2	13 (45%)
Tumor differentiation, *n* (%)	
Well/Moderate	16 (60%)
Poor	8 (27%)
Unknown	5 (13%)
Tumor size (cm)	
Median (IQR)	2.9 (2.4-4.0)
Tumor location, *n* (%)	
Head	22 (76%)
Body/tail	7 (24%)

Abbreviations: IQR, *interquartile range*; CA19-9, *carbohydrate antigen 19-9*; COPD, *chronic obstructive pulmonary disease*; SBRT, *stereotactic body radiation therapy*; ECOG PS, *Eastern Cooperative Oncology Group performance status*.

Treatment characteristics are summarized in Table 2. Most patients (*n*=22, 76%) received induction chemotherapy prior to beginning SBRT for a median duration of 2 months (IQR, 1-4). Induction chemotherapy regimens varied, with most patients receiving gemcitabine-based (as a single agent (*n*=7, 24%) or with either nab-paclitaxel or capecitabine and docetaxel (*n*=10, 34%)) or 5-fluorouracil-based (5-fluorouracil, leucovorin, irinotecan, and oxaliplatin [FOLFIRINOX] (*n*=5, 17%)) chemotherapy and 76% of patients receiving a multi-agent regimen. The median time interval from diagnosis to start of induction chemotherapy was 2 weeks (IQR, 2-4 weeks). SBRT was delivered over 5 fractions to a median dose of 28 Gy (IQR, 25-33 Gy). The median time interval from diagnosis to start of SBRT was 5.5 months (IQR, 2-6 months). After SBRT, 9 patients (31%) received maintenance chemotherapy for a median duration of 8 months (IQR, 2-8.5 months). All maintenance chemotherapy regimens consisted of gemcitabine (as a single agent or with either nab-paclitaxel or capecitabine). The median time interval from completion of SBRT to start of maintenance chemotherapy was 2 weeks. There was overlap between patients who received induction chemotherapy and maintenance chemotherapy, as 7 patients received both induction and maintenance chemotherapy, while 15 received induction chemotherapy alone and 2 received maintenance chemotherapy alone. Five patients received no chemotherapy before or after SBRT. None of the 29 patients received any other local tumor-directed therapy prior to or after SBRT.

**Table 2:** Treatment characteristics

*Treatment characteristics*	*All patients (n=29)*
SBRT tumor motion control, *n* (%)	
Active breathing control	17 (63%)
Free breathing	12 (37%)
SBRT dose (Gy)	
Median total dose (IQR)	28 (25-33)
Median dose per fraction (IQR)	5.8 (5-6.6)
Systemic therapy, *n* (%)	
Induction CTX before SBRT only	15 (52%)
Maintenance CTX after SBRT only	2 (7%)
Both induction CTX and maintenance CTX	7 (24%)
No CTX received	5 (17%)

Abbreviations: IQR, *interquartile range*; SBRT, *stereotactic body radiation therapy*; CTX, *chemotherapy*; Gy, *gray*.

### Survival

**Figure 1:**
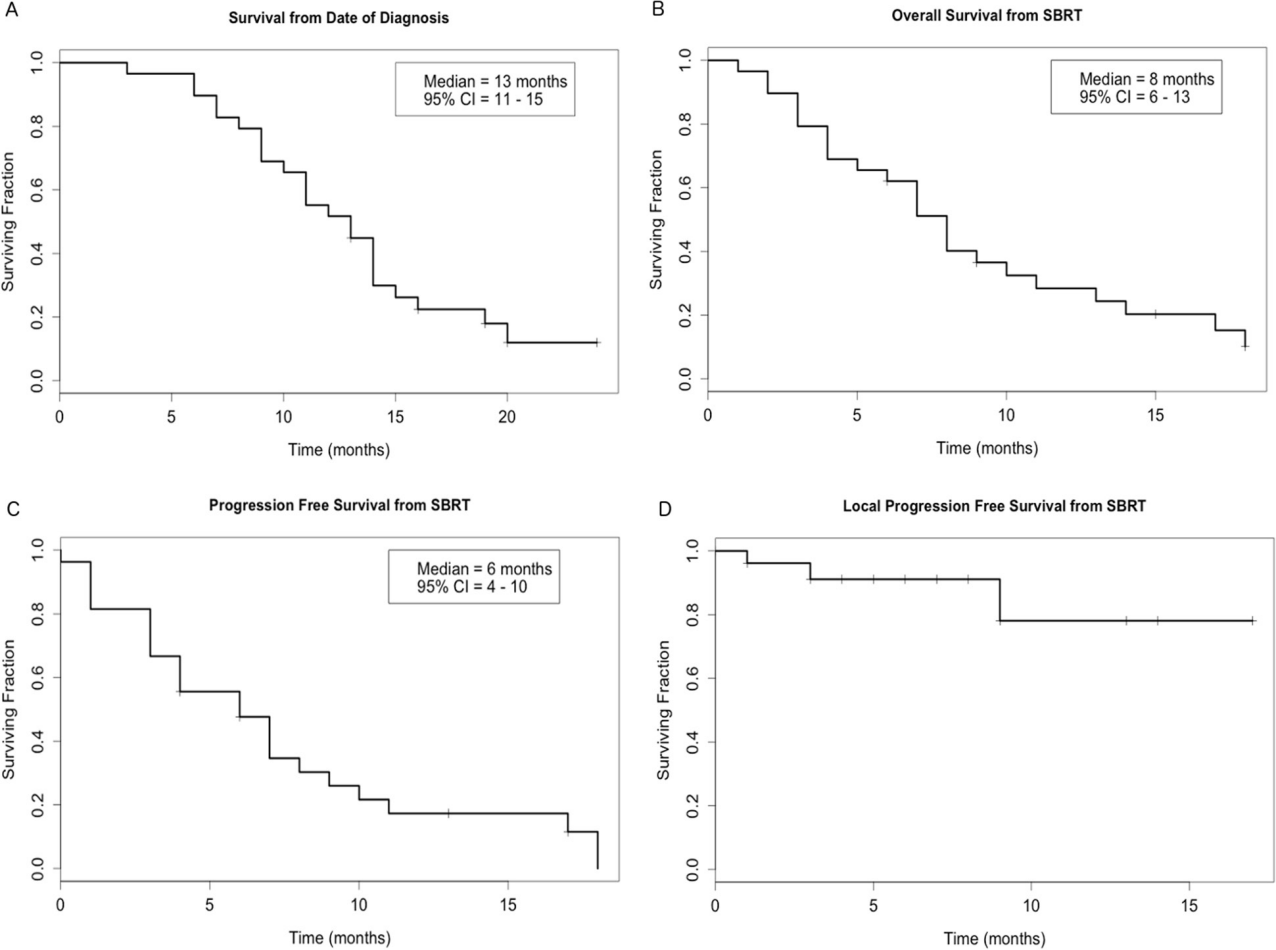
Kaplan-Meier curves showing **(A)** survival from the date of histologic diagnosis and **(B)** overall survival, **(C)** progression-free survival, and **(D)** local progression-free survival from the date of the first fraction of stereotactic body radiation therapy (SBRT). CI indicates confidence interval.

At the time of analysis, 24 patients (83%) had died, and 5 patients (17%) were alive and censored after a median of 15 months of follow-up (IQR, 4-18). Median overall survival (mOS) was 13 months from the date of histologic diagnosis (95% confidence interval [CI]: 11-15) and 8 months from the first day of SBRT (95% CI: 6-13) (Figure 1A-1B). Six- and 12-month rates of OS were 89% and 52% from the date of histologic diagnosis and 62% and 28% from the first day of SBRT. Median progression-free survival (mPFS) from the first day of SBRT was 6 months, and 6- and 12-month rates of PFS were 48% and 17% respectively (Figure 1C). The sites of first distant metastasis were liver (*n* = 9), lungs (*n* = 3), and brain (*n* = 1). During follow-up, 3 patients experienced local progression based on imaging, yielding 6- and 12-month local PFS (LPFS) rates of 91% and 78%, respectively (Figure 1D). Median LPFS was not reached.

**Figure 2:**
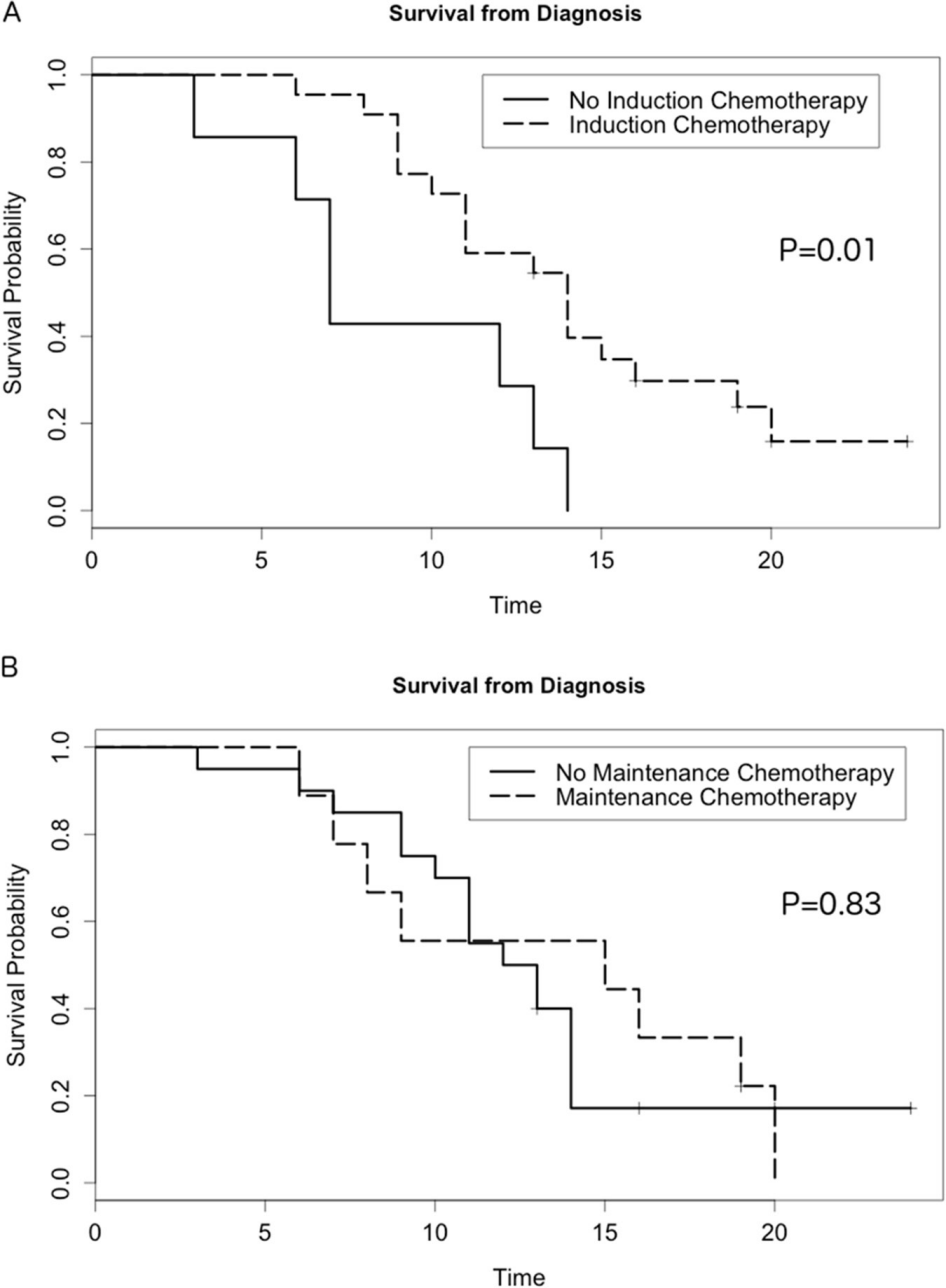
Kaplan-Meier curves showing overall survival from the date of diagnosis stratified by **(A)** administration of induction chemotherapy prior to SBRT and **(B)** administration of maintenance chemotherapy after SBRT.

To assess the importance of systemic therapy, survival from the date of diagnosis was compared between patients who received induction chemotherapy prior to SBRT and patients who did not. Patients receiving induction chemotherapy had superior survival than those who did not (14 vs. 7 months, p = 0.01) (Figure 2A). There was not a significant difference in survival between patients who received maintenance chemotherapy after SBRT and those who did not (15 vs. 12 months, p = 0.83) (Figure 2B).

### Symptom palliation and performance status

Symptom palliation was achieved within 3 months in 8 of 11 patients (73%) experiencing abdominal pain, 3 of 15 patients (20%) experiencing fatigue, 7 of 12 patients (58%) experiencing anorexia, 8 of 10 patients (80%) experiencing weight loss, and 5 of 5 patients (100%) experiencing nausea (Table 3). Of the 8 patients who experienced weight loss whose weight stabilized or improved after SBRT, the average weight change was an increase of 2.86 kg within 3 months after SBRT. Of the 13 patients who had an ECOG PS of 2 prior to SBRT, 3 (23%) had an improvement of ECOG PS to 0 or 1 at 3-month follow-up, and 1 of the 16 patients (6%) with an ECOG PS of 0 or 1 prior to SBRT had a decline of ECOG PS to 2 at 3-month follow-up.

**Table 3:** Summary of symptom palliation

Symptom	Abdominal Pain	Fatigue	Anorexia	Weight loss	Nausea
Pre-SBRT symptoms, *n*	11	15	12	10	5
Improvement after SBRT, *n*	8	3	7	8	5
Percentage improved	73%	20%	58%	80%	100%

Abbreviation: SBRT, *stereotactic body radiation therapy*.

### Toxicity

Of the 29 patients included, 12 experienced acute toxicities of grade ≥2. The total number of patients experiencing acute toxicity and the types of toxicities experienced (some patients experienced more than one type) are detailed in Table 4. None experienced acute enteritis, fistula, gastritis, or ulcer of grade ≥2. One patient experienced a grade 3 gastrointestinal bleed 1 month after completing SBRT that was possibly attributed to treatment. The patient required transfusion of blood products after which the bleeding resolved. The source of the bleed was not determined. Two patients experienced grade 3 abdominal pain. No acute grade 4 or 5 toxicity was observed.

**Table 4:** Acute and late gastrointestinal toxicities presented by time frame, type, and severity^a^

Category	Grade 2 (%)	Grade 3 (%)	Grade 4 (%)	Grade 5 (%)
Acute toxicity (*n*=12)				
Enteritis	0 (0)	0 (0)	0 (0)	0 (0)
Fistula	0 (0)	0 (0)	0 (0)	0 (0)
Gastritis	0 (0)	0 (0)	0 (0)	0 (0)
Ulcer	0 (0)	0 (0)	0 (0)	0 (0)
Fatigue	8 (28)	0 (0)	0 (0)	0 (0)
Abdominal Pain	7 (24)	2 (7)	0 (0)	0 (0)
Anorexia	4 (14)	0 (0)	0 (0)	0 (0)
Nausea	3 (10)	0 (0)	0 (0)	0 (0)
Vomiting	1 (3)	0 (0)	0 (0)	0 (0)
Constipation	1 (3)	0 (0)	0 (0)	0 (0)
Dehydration	0 (0)	0 (0)	0 (0)	0 (0)
Diarrhea	1 (3)	0 (0)	0 (0)	0 (0)
Weight Loss	0 (0)	0 (0)	0 (0)	0 (0)
Other	0 (0)	1 (3)^b^	0 (0)	0 (0)
Late toxicity (*n*=1)				
Enteritis	0 (0)	0 (0)	0 (0)	0 (0)
Fistula	0 (0)	0 (0)	0 (0)	0 (0)
Gastritis	0 (0)	0 (0)	1 (2)	0 (0)
Ulcer	0 (0)	0 (0)	0 (0)	0 (0)
Other	0 (0)	0 (0)	0 (0)	0 (0)

^a^ Toxicity was assessed using the National Cancer Institute Common Terminology for Adverse Events [version 4.0].

^b^ Gastrointestinal bleed of unidentified source requiring transfusion of blood products.

Late toxicity data were only available for 26 patients (90%) because 3 patients died within 3 months of SBRT. Of these 3 patients who died within 3 months of SBRT, one died 2 months after SBRT after presenting to the hospital with acute kidney injury and severe hypernatremia credited to multifactorial dehydration. Cause of death was not definitively determined for the other two patients, but both had evidence of metastatic lesions in the liver on follow-up CT suggesting that metastatic disease was a likely cause of death. Of the 26 patients surviving for more than 3 months, 1 patient (4%) experienced late grade 4 enterocutaneous fistula requiring emergent surgical intervention. No other late grade ≥2 toxicity was observed.

## DISCUSSION

The optimal treatment strategy for patients with localized PDAC who cannot tolerate surgical treatment due to comorbid conditions, poor performance status, and/or advanced age has not been clearly defined. While most clinical trials for localized PDAC focus on patients who can tolerate aggressive multi-modality therapy including surgery, chemotherapy, and/or radiation, it is important to find treatment options for patients who cannot tolerate existing therapies, especially considering the prevalence of debilitating symptoms associated with PDAC and comorbid conditions in elderly patients [3, 4].

Treatment of PDAC in the elderly has been reported in several studies. In a series of 68 patients aged 65 and older, Matsumoto et al. found that low-dose gemcitabine may provide better survival from diagnosis than best supportive care alone (median 7.6 vs. 2.3 months, p<0.0001) [6]. Despite the systemic benefit of low-dose chemotherapy in these patients, autopsy studies have demonstrated that locally destructive tumor growth is the probable cause of death in up to 30% of patients with PDAC [16]. These data suggest that, if tolerated, local therapy could be of significant benefit to patients either in conjunction with systemic therapy or alone. Horowitz et al. studied the outcomes of 166 patients aged 75 or older who underwent surgical resection for PDAC [17]. Reported mOS was 22 months for patients who received adjuvant chemoradiation after surgery and 14 months for patients who received surgery alone. While this study demonstrates that some elderly patients may benefit from surgical resection, performance status and comorbidity data were not reported, thereby making it difficult to generalize these results to all elderly patients with PDAC. Further, study of surgical outcomes has demonstrated that mortality risk and length of post-surgical hospital stay increases proportionally with increasing age, and elderly patients are at an increased risk for complications following surgery including delayed gastric emptying, pancreatic fistulas, and neurologic sequelae [18–21]. Miyamoto et al. reported the outcomes of 42 patients aged 75 or older who received chemoradiation therapy for PDAC [22]. Of these 42 patients, 24 received definitive chemoradiation therapy for locally advanced pancreatic cancer and 18 had disease treated with surgery and chemoradiation. Among those treated with definitive chemoradiation alone (and not surgery), mOS was 8.6 months (95% CI: 7.2-13.1) but toxicity rates were high with 29% of patients being hospitalized during therapy, 54% requiring treatment breaks, and 33% unable to complete therapy. While acute toxicity rates were considerable, this study did not report late toxicity rates. These studies demonstrate that although aggressive therapy may benefit some elderly patients, unwanted treatment-related complications and toxicity rates remain high in this population with limited expected survival outlook. Further, the question still remains of how to best manage elderly patients who cannot tolerate these aggressive therapies.

SBRT is a promising local therapy that has been studied as an alternative to conventionally fractionated radiation therapy. Zhong et al. compared the outcomes of 7,819 patients treated with conventionally fractionated radiation therapy to 631 patients treated with SBRT [23]. Treatment with SBRT was associated improved OS on multivariate analysis (HR = 0.84, 95% CI = 0.75 – 0.93). In a propensity-matched analysis, mOS (13.9 vs. 11.6 months) and the two-year OS rates (21.7% vs. 16.5%) were significantly higher with SBRT than conventionally fractionated radiation therapy. In a study of 44 patients treated with SBRT and 226 patients treated with intensity modulated radiation therapy (IMRT), SBRT was associated with lower rates of grade 2 and 3 acute toxicities than IMRT, although no difference in OS was found [24]. In addition to possibly improved survival and lower rates of toxicity, SBRT can be delivered in only 5 fractions, while conventionally fractionated radiation therapy can take more than 20 days. In a patient population with limited life expectancy, a shorter duration of treatment is a significant consideration and could improve quality of life.

SBRT has shown promise in small studies as a local therapy option for patients who cannot tolerate surgery or chemoradiation. Yechieli et al. reported the outcomes of 20 patients deemed medically inoperable who were treated with SBRT [14]. Seventeen patients had an Adult Comorbidity Evaluation-27 score of moderate or severe, 60% of patients had and ECOG PS of 2-3, and the mean age was 83 (range 77-90). Three patients experienced a grade 3 or 4 treatment-related toxicity. mOS was 6.4 months (95% CI: 3.5-10.8). Median recurrence-free survival was 6.8 months (95% CI: 1.3-23.5). Kim et al. reported the outcomes of 26 elderly patients aged ≥80 who were treated with SBRT [15]. mOS was 7.6 months, and median local control was 11.5 months. There were no acute or late grade ≥3 treatment-related toxicities. These studies suggest the potential for SBRT to safely treat PDAC in patients who cannot tolerate more aggressive therapy.

This study includes patients who were deemed incapable of tolerating surgical therapy by a multidisciplinary oncology team. Median age was 74, 45% of patients had an ECOG PS of 2, and rates of comorbid conditions were high. Despite the presence of advanced age, comorbid conditions, and poor performance status, most patients tolerated SBRT well with low rates of grade ≥2 acute and late toxicity. Only 3 of 29 patients (10%) experienced acute grade ≥3 toxicity, and 1 of 26 patients (4%) experienced late grade ≥3 toxicity. The favorable toxicity profile observed in this study may be related in part to the dose of SBRT delivered (median 28 Gy, IQR 25-33), which was lower than that reported in some other studies that reported favorable rates of local control [25].

Clinical outcomes were encouraging in our study with a median survival of 13 months from the date of diagnosis and 8 months from the start of SBRT. SBRT offered good local control with 6 and 12-month rates of 91% and 78%, respectively. With low rates of toxicity and encouraging local disease control, SBRT could be preferred over palliative chemotherapy alone or best supportive care in patients with localized disease who cannot undergo surgical resection. Of note, although the patients included in our study were deemed unable to tolerate surgery, they were generally younger (median age 74, IQR 68-79) and had a better performance status (45% ECOG 2) compared to the patients in the studies by Kim et al. and Yechieli et al. In addition, 22 patients (76%) in our study received induction chemotherapy before SBRT and only 5 patients (17%) did not receive any chemotherapy before or after SBRT. By comparison, in the study by Kim et al. only 4 patients (15%) received induction chemotherapy, 6 patients (23%) received maintenance chemotherapy, and 16 (62%) did not receive any chemotherapy. In the study by Yechieli et al., no patients received chemotherapy before SBRT, and only one (5%) received maintenance capecitabine after SBRT. Therefore, the favorable survival outcomes observed in our study in comparison to those reported by Kim et al. and Yechieli et al. may be related in part to differences in baseline patient characteristics, such as younger age and improved performance status, and to the increased use of systemic chemotherapy.

In fact, in our study patients receiving induction chemotherapy prior to SBRT had superior survival outcomes than patients who did not receive induction chemotherapy before SBRT. To the authors’ knowledge, this is the first study to demonstrate that induction chemotherapy before SBRT is associated with superior survival than SBRT without induction chemotherapy in elderly, medically inoperable patients. There was no survival difference between patients who received maintenance chemotherapy after SBRT and those who did not. These findings suggest that the combination of induction chemotherapy and SBRT may provide systemic and local control respectively, which are both of great importance as systemic disease and local progression account for approximately 70% and 30% of PDAC related deaths respectively [16]. However, given the nonrandomized nature of systemic and local treatment choices in our study, it is difficult to draw conclusions with certainty about the benefits and ideal timing of systemic chemotherapy in relation to SBRT, and future prospective studies are warranted.

In the current study, patients experienced high rates of symptom palliation after receiving SBRT, especially patients who experienced abdominal pain and nausea prior to the start of treatment. The results also suggest that SBRT may be associated with an improvement or at least a stabilization of performance status. For patients who are elderly, suffering from comorbid conditions, and/or have a poor performance status, quality of life is an important consideration in choosing between treatment options. The capacity of SBRT to improve patients’ symptoms and the relatively short period of SBRT delivery (as few as five days) make this treatment modality an especially attractive option for patients prioritizing quality of life. Although SBRT generally requires an endoscopic procedure for fiducial placement, it is less invasive and better tolerated than surgery.

This study is limited primarily by its retrospective nature. Thus, variations in treatment (including systemic therapy) were non-random, making it difficult to determine the importance of different variables in determining patients’ outcomes. Future prospective studies focusing on patient-reported outcomes are warranted to further elucidate the impact of SBRT on the quality of life in this unique patient population and to further define the importance of chemotherapy before and after SBRT.

In conclusion, the use of SBRT to treat patients unable to tolerate surgical resection due to poor performance status, advanced age, and/or comorbid conditions appears to be safe and effective. The observed median survival of 13 months from diagnosis is favorable. Observed rates of treatment-related acute and late toxicity were acceptable, and patients experienced significant improvement of symptoms after treatment. Patients may have superior survival outcomes if they receive induction chemotherapy before SBRT. While this study demonstrates SBRT is a viable treatment option even in the setting of severe comorbidities, poor performance status, and advanced age, future studies are warranted to further elucidate the importance of chemotherapy in relation to SBRT and to assess the impact of SBRT on patient reported measures of quality of life.

## MATERIALS AND METHODS

### Patient selection

With the approval of our Institutional Review Board, we retrospectively reviewed the records of all PDAC patients treated with SBRT at our institution between 2010 and 2016. Patients who were unable to undergo curative intent surgery as determined by a multidisciplinary oncology team including medical oncologists, surgical oncologists, radiation oncologists, pathologists, and radiologists due to medical comorbidities, poor performance status (ECOG PS ≥2), advanced age (≥70 years), or a combination of these factors were included. All patients underwent fine needle aspiration (FNA) to histologically confirm the diagnosis of PDAC. Patients with radiographic evidence of metastatic disease prior to delivery of SBRT were excluded. Patients had serial follow-up every 3-6 months after SBRT including imaging with pancreas protocol computed tomography (CT) of the chest, abdomen and pelvis.

### SBRT planning and delivery

Patients underwent a simulation CT scan with oral or intravenous contrast while in the supine position in a custom-made immobilization device. Using the simulation scan, a radiation oncologist defined the gross tumor volume (GTV). Tumor motion during respiration was measured using a 4-dimensional CT scan. If tumor motion exceeded 3-mm, active breathing control (ABC) was used during treatment (*n*=17, 59%). Patients were treated at end inspiration. If tumor motion was less than 3-mm or patient could not tolerate ABC, an internal target volume (ITV) was defined after review of diagnostic CT, respiration-correlated 4-dimensional CT, pancreas protocol CT, and positron emission tomography (PET)/CT scans when available (*n*=12, 41%). The planning target volume (PTV) was then defined by a 1- to 3-mm margin expansion of the GTV or ITV depending on the distance of the tumor from the adjacent duodenum, small bowel, or stomach. Initial patient positioning was based on cone-beam CT with alignment to spine. Volumetric kV-imaging was then used to align biliary stents (*n*=14, 48%) and/or fiducials (*n*=25, 86%) for treatment delivery. Three patients (10%) were aligned to spine alone due to the inability to place fiducials. SBRT was delivered with a linear accelerator based approach in all cases.

### Clinical outcomes

Data concerning patient and treatment characteristics, symptom palliation, treatment toxicity, and survival were obtained from patient charts. The date of progression was defined as the date of the first follow-up cross-sectional imaging study (CT or PET/CT) demonstrating local progression or distant metastasis. Distant and local progression were determined using RECIST (Response Evaluation Criteria in Solid Tumors) version 1.1 guidelines. Survival was measured from the date of histologic diagnosis until the date of death, censoring patients at the date of last follow-up if no date of death was available. OS, PFS, and LPFS from SBRT were measured from the date of the first fraction of SBRT until the date of death, first progression (local or distant), or local progression, respectively, censoring patients at the date of last follow-up (OS) or last CT scan (PFS and LPFS). To assess the presence of common comorbidities, patients’ records were examined for a history of chronic obstructive pulmonary disease (diagnosed and staged using the Global Initiative for Chronic Obstructive Lung Disease (GOLD) diagnostic criterion), cardiovascular disease (including myocardial infarction, coronary artery disease, congestive heart failure, or arrhythmias of grade ≥2 [moderately to severely decompensated] staged using Adult Comorbidity Evaluation-27 criteria), diabetes mellitus, hypertension, and history of another cancer. Patients were assigned an Adult Comorbidity Evaluation-27 score based on their comorbid conditions. Symptoms evaluated using the National Cancer Institute Common Terminology Criteria for Adverse Events (NCI CTCAE) prior to the start of SBRT were abdominal pain, fatigue, weight loss (defined as loss of ≥10% of baseline weight), anorexia, and nausea [26]. Effective palliation of abdominal pain, fatigue, anorexia, or nausea was defined as improvement of the NCI CTCAE score relative to the pretreatment score at 3-month follow up. Improvement of weight loss was considered to be stabilization or increase of weight from pre-SBRT weight at 3-month follow up. Significant gastrointestinal treatment-related toxicities were evaluated using the NCI CTCAE. Acute toxicities were those occurring within 3 months of SBRT, and late toxicities were those occurring more than 3 months after SBRT.

### Statistical analysis

Demographic, clinicopathologic, and treatment characteristics were summarized using descriptive statistics. Survival outcomes were estimated using Kaplan-Meier curves. Associations between survival and receiving induction or maintenance chemotherapy were evaluated with the log-rank test. A two-sided alpha level of ≤0.05 was considered statistically significant. Statistical analyses were performed using R version 3.1.2 (R Foundation for Statistical Computing, Vienna, Austria).

## References

[1] Siegel RL, Miller KD, Jemal A (2016). Cancer statistics, 2016. CA Cancer J Clin.

[2] Rosati LM, Herman JM (2017). Role of Stereotactic Body Radiotherapy in the Treatment of Elderly and Poor Performance Status Patients With Pancreatic Cancer. J Oncol Pract.

[3] National Cancer Institute SEER Statistics Fact Sheets: Pancreatic Cancer. https://seer.cancer.gov/statfacts/html/pancreas.html.

[4] Higuera O, Ghanem I, Nasimi R, Prieto I, Koren L, Feliu J (2016). Management of pancreatic cancer in the elderly. World J Gastroenterol.

[5] Hsu CC, Wolfgang CL, Laheru DA, Pawlik TM, Swartz MJ, Winter JM, Robinson R, Edil BH, Narang AK, Choti MA, Hruban RH, Cameron JL, Schulick RD (2012). Early mortality risk score: identification of poor outcomes following upfront surgery for resectable pancreatic cancer. J Gastrointest Surg.

[6] Matsumoto K, Miyake Y, Kato H, Kawamoto H, Imagawa A, Toyokawa T, Nakatsu M, Ando M, Hirohata M, Yamamoto K (2011). Effect of low-dose gemcitabine on unresectable pancreatic cancer in elderly patients. Digestion.

[7] Chang DT, Schellenberg D, Shen J, Kim J, Goodman KA, Fisher GA, Ford JM, Desser T, Quon A, Koong AC (2009). Stereotactic radiotherapy for unresectable adenocarcinoma of the pancreas. Cancer.

[8] Herman JM, Chang DT, Goodman KA, Dholakia AS, Raman SP, Hacker-Prietz A, Iacobuzio-Donahue CA, Griffith ME, Pawlik TM, Pai JS, O’Reilly E, Fisher GA, Wild AT (2015). Phase 2 multi-institutional trial evaluating gemcitabine and stereotactic body radiotherapy for patients with locally advanced unresectable pancreatic adenocarcinoma. Cancer.

[9] Koong AC, Le QT, Ho A, Fong B, Fisher G, Cho C, Ford J, Poen J, Gibbs IC, Mehta VK, Kee S, Trueblood W, Yang G, Bastidas JA (2004). Phase I study of stereotactic radiosurgery in patients with locally advanced pancreatic cancer. Int J Radiat Oncol Biol Phys.

[10] Koong AC, Christofferson E, Le QT, Goodman KA, Ho A, Kuo T, Ford JM, Fisher GA, Greco R, Norton J, Yang GP (2005). Phase II study to assess the efficacy of conventionally fractionated radiotherapy followed by a stereotactic radiosurgery boost in patients with locally advanced pancreatic cancer. Int J Radiat Oncol Biol Phys.

[11] Didolkar MS, Coleman CW, Brenner MJ, Chu KU, Olexa N, Stanwyck E, Yu A, Neerchal N, Rabinowitz S (2010). Image-guided stereotactic radiosurgery for locally advanced pancreatic adenocarcinoma results of first 85 patients. J Gastrointest Surg.

[12] Schellenberg D, Goodman KA, Lee F, Chang S, Kuo T, Ford JM, Fisher GA, Quon A, Desser TS, Norton J, Greco R, Yang GP, Koong AC (2008). Gemcitabine chemotherapy and single-fraction stereotactic body radiotherapy for locally advanced pancreatic cancer. Int J Radiat Oncol Biol Phys.

[13] Schellenberg D, Kim J, Christman-Skieller C, Chun CL, Columbo LA, Ford JM, Fisher GA, Kunz PL, Van Dam J, Quon A, Desser TS, Norton J, Hsu A (2011). Single-fraction stereotactic body radiation therapy and sequential gemcitabine for the treatment of locally advanced pancreatic cancer. Int J Radiat Oncol Biol Phys.

[14] Yechieli RL, Robbins JR, Mahan M, Siddiqui F, Ajlouni M (2017). Stereotactic body radiotherapy for elderly patients with medically inoperable pancreatic cancer. Am J Clin Oncol.

[15] Kim CH, Ling DC, Wegner RE, Flickinger JC, Heron DE, Zeh H, Moser AJ, Burton SA (2013). Stereotactic body radiotherapy in the treatment of pancreatic adenocarcinoma in elderly patients. Radiat Oncol.

[16] Iacobuzio-Donahue CA, Fu B, Yachida S, Luo M, Abe H, Henderson CM, Vilardell F, Wang Z, Keller JW, Banerjee P, Herman JM, Cameron JL, Yeo CJ (2009). DPC4 gene status of the primary carcinoma correlates with patterns of failure in patients with pancreatic cancer. J Clin Oncol.

[17] Horowitz DP, Hsu CC, Wang J, Makary MA, Winter JM, Robinson R, Schulick RD, Cameron JL, Pawlik TM, Herman JM (2011). Adjuvant chemoradiation therapy after pancreaticoduodenectomy in elderly patients with pancreatic adenocarcinoma. Int J Radiat Oncol Biol Phys.

[18] Riall TS, Sheffield KM, Kuo YF, Townsend CM, Goodwin JS (2011). Resection benefits older adults with locoregional pancreatic cancer despite greater short-term morbidity and mortality. J Am Geriatr Soc.

[19] Finlayson E, Fan Z, Birkmeyer JD (2007). Outcomes in octogenarians undergoing high-risk cancer operation: a national study. J Am Coll Surg.

[20] Ito Y, Kenmochi T, Irino T, Egawa T, Hayashi S, Nagashima A, Kitagawa Y (2011). The impact of surgical outcome after pancreaticoduodenectomy in elderly patients. World J Surg Oncol.

[21] Hodul P, Tansey J, Golts E, Oh D, Pickleman J, Aranha GV (2001). Age is not a contraindication to pancreaticoduodenectomy. Am Surg.

[22] Miyamoto DT, Mamon HJ, Ryan DP, Willett CG, Ancukiewicz M, Kobayashi WK, Blaszkowsky L, Fernandez-del Castillo C, Hong TS (2010). Outcomes and tolerability of chemoradiation therapy for pancreatic cancer patients aged 75 years or older. Int J Radiat Oncol Biol Phys.

[23] Zhong J, Patel K, Switchenko J, Cassidy RJ, Hall WA, Gillespie T, Patel PR, Kooby D, Landry J (2017). Outcomes for patients with locally advanced pancreatic adenocarcinoma treated with stereotactic body radiation therapy versus conventionally fractionated radiation. Cancer.

[24] Park JJ, Hajj C, Reyngold M, Shi W, Zhang Z, Cuaron JJ, Crane CH, O’Reilly EM, Lowery MA, Yu KH, Goodman KA, Wu AJ (2017). Stereotactic body radiation vs. intensity-modulated radiation for unresectable pancreatic cancer. Acta Oncol.

[25] Brunner TB, Nestle U, Grosu AL, Partridge M (2015). SBRT in pancreatic cancer: what is the therapeutic window?. Radiother Oncol.

[26] National Cancer Institute Common Terminology Criteria for Adverse Events (CTCAE). Version 4.0. https://ctep.cancer.gov/protocoldevelopment/electronic_applications/ctc.htm.

